# Community Composition and Year-round Abundance of Vector Species of Mosquitoes make Miami-Dade County, Florida a Receptive Gateway for Arbovirus entry to the United States

**DOI:** 10.1038/s41598-019-45337-2

**Published:** 2019-06-19

**Authors:** André B. B. Wilke, Chalmers Vasquez, Johana Medina, Augusto Carvajal, William Petrie, John C. Beier

**Affiliations:** 1 Department of Public Health Sciences, Miller School of Medicine, University of Miami, Miami, FL United States of America; 2 Miami-Dade County Mosquito Control Division, Miami, FL United States of America

**Keywords:** Urban ecology, Community ecology

## Abstract

Vector-borne diseases are a heavy burden to human-kind. Global warming and urbanization have a significant impact on vector-borne disease transmission, resulting in more severe outbreaks, and outbreaks in formerly non-endemic areas. Miami-Dade County, Florida was the most affected area in the continental United States during the 2016 Zika virus outbreak. Miami is an important gateway and has suitable conditions for mosquitoes year-round. Therefore, it was critical to establish and validate a surveillance system to guide and improve mosquito control operations. Here we assess two years of mosquito surveillance in Miami established after the 2016 Zika virus outbreak. Our results show that the most abundant mosquito species are either well adapted to urban environments or are adapting to it. The five most abundant species comprised 85% of all specimens collected, with four of them being primary vectors of arboviruses. *Aedes aegypti* and *Culex quinquefasciatus* were found year-round throughout Miami regardless of urbanization level, vegetation, or socioeconomic variations. This study serves as a foundation for future efforts to improve mosquito surveillance and control operations.

## Introduction

Vector-borne diseases (VBDs) affect more than half of all human populations living in endemic areas of the globe^1^. Current estimates show that dengue virus (DENV) infects around 390 million people every year^2^. The Pan American Health Organization (PAHO) officially confirmed 1,003,509 cases of Zika virus (ZIKV) between 2015 and 2018 in the Americas^3^, and subsequent studies have also shown the increase in fetus malformation with ZIKV infection during pregnancy^4,5^.

Considerable efforts have been allocated to fight vector mosquitoes. However, the efforts to control mosquito populations have only achieved limited success, and the global incidence of VBDs is currently on the rise^6–9^. Not only have more severe VBD outbreaks been reported but outbreaks have occurred in formerly non-endemic countries such as Italy, France and Croatia^10–13^. Furthermore, several arboviruses are circulating in tropical regions of the world, and many more are circulating under the radar^14–19^.

There are only limited options for the treatment of arbovirus infections and their prevention by vaccination. Therefore, controlling vector mosquito populations is widely accepted as the most effective way to prevent the transmission of VBDs^20^. Controlling vector mosquitoes rely on many steps that logically build on each other. Effective surveillance is fundamental as mosquitoes are often locally concentrated, abundant, and harder to control primarily in those specific, definable habitats at the neighborhood level. These favorable habitats provide optimal conditions and environmental resources needed for mosquito survival, a key determinant of their vectorial capacity. It is critical for the development of any mosquito control strategy to know through effective surveillance the geographic distribution, community composition and relative abundance of vector mosquitoes as well as the risk of introduction of arboviruses^21,22^.

Miami-Dade County, Florida was the most affected area in the continental United States during the 2016 Zika virus outbreak^23^. Miami is not only one of the most important gateways to the U.S. with an increased flow of people coming and going from endemic areas, but its proximity to the Caribbean region and Latin America substantially increases the risk of introduction of arboviruses to the U.S. Moreover, Miami also has the appropriate conditions for mosquitoes, its climate is defined as tropical monsoon^24^, and is conducive for mosquitoes even during the winter. Miami is also undergoing an intense increase in urbanization^25^ that impacts the population dynamics of vector mosquitoes and patterns of VBD transmission^26^.

Historically, Miami-Dade County and, in a broader perspective, South Florida have been afflicted by arbovirus outbreaks for decades, including DENV, West Nile virus (WNV) and YFV^27–32^. However, as seen during the most recent ZIKV outbreak, the virus was introduced in Miami multiple times on different occasions^33^, exposing the real vulnerability of Miami to the introduction of arboviruses and subsequent VBD outbreaks.

Therefore, it was critical to establish a state-of-the-art surveillance system to determine the community composition and abundance of mosquitoes in Miami-Dade County, Florida, to help inform, guide and improve mosquito control operations. Here our objective was to assess the last two years of mosquito surveillance data in Miami-Dade County, Florida.

## Results

### Mosquito composition and abundance

A total of 2,711,983 mosquitoes were collected in Miami-Dade County from August 2016 to November 2018 by the 157 BG-Sentinel and 34 CDC traps. The collected mosquitoes comprised 41 species from 9 genera. The most abundant species was *Culex nigripalpus* comprising 1,057,485 (38%) specimens collected, followed by *Aedes taeniorhynchus* 626,163 (23%), *Culex quinquefasciatus* 373,571 (13%), *Aedes aegypti* 150,588 (5%) and *Anopheles crucians* 132,741 (4%). These 5 species comprised 85% of all collected specimens.

BG-Sentinel traps collected a total of 568,565 mosquitoes, from which 355,381 were *Cx. quinquefasciatus* (62%) and 134,652 *Ae. aegypti* (23%), comprising 85% of all collected mosquitoes. CDC traps collected a total of 2,143,418 mosquitoes, from which 1,034,119 were *Cx. nigripalpus* (48%), 610,547 *Ae. taeniorhynchus* (28%), 131,077 *An. crucians* (6%) and 95,193 *Aedes tortilis* (4%), comprising 85% of all collected mosquitoes. BG-Sentinel traps did not collect *Aedes fulvuspallens*, *Aedes scapularis*, *Anopheles walker*, *Culex bahamensis*, *Culex cedecei*, *Psorophora howardii* and *Psorophora johnstonii*. CDC traps failed to collect *Culex biscaynensis* (Table 1).

**Table 1:** Mosquito species collected in Miami-Dade County from May 2016 to November 2018.

Species	CDC	BG-Sentinel	Total	Epidemiological Importance
*Aedes* (*Stegomyia*) *aegypti* (Linnaeus, 1762)	15,936	134,652	150,588	CHIKV^20,68^, DENV^68,69^, MAYV^70^, OROV^68^, YFV^68,71^, WNV^72^, ZIKV^68,73^
*Aedes* (*Stegomyia*) *albopictus* (Skuse, 1895*)*	11,405	808	12,213	CHIKV^20^, DENV^20^, YFV^60,74^, WNV^72^, ZIKV^73^
*Aedes* (*Ochlerotatus*) *atlanticus* (Dyar & Knab, 1906)	48,538	81	48,619	CALV^75^, EEEV^68^, KEYV^76^, WNV^72^
*Aedes* (*Howardina*) *bahamensis* (Berlin, 1969)	1,447	8	1,455	SLE^77^
*Aedes* (*Ochlerotatus*) *fulvus* (Wiedemann, 1828)	28	0	28	EEEV^68^, YFV^78^, WNV^72^
*Aedes* (*Ochlerotatus*) *infirmatus* (Dyar & Knab, 1906)	8,582	4	8,586	EEEV^68^, KEYV^68^, WNV^72^
*Aedes* (*Ochlerotatus*) *scapularis* (Rondani, 1848)	131	0	131	MAYV, OROV^68^, ROCV^79^, YFV^80^
*Aedes* (*Ochlerotatus*) *taeniorhynchus* (Wiedemann, 1821)	610,547	15,616	626,163	EEEV^68^, EVEV^81^, KEYV^68^, WNV^72^, ZIKV^68^
*Aedes* (*Ochlerotatus*) *tortilis* (Theobald, 1903)	95,193	7,333	102,526	Unknown
*Aedes* (*Ochlerotatus*) *triseriatus* (Say, 1823)	2,538	632	3,170	KEYV^68^, ZIKV^68^
*Anopheles* (*Nyssorhynchus*) *albimanus* (Wiedemann, 1820)	120	2	122	Malaria^82^
*Anopheles* (*Anopheles*) *atropos* (Dyar & Knab, 1906)	5,017	114	5,131	WNV^72^
*Anopheles* (*Anopheles*) *crucians* (Wiedemann, 1828)	131,077	1,664	132,741	EEEV^68^, WNV^83^
*Anopheles* (*Anopheles*) *punctipennis* (Martini, 1932)	121	4	125	Malaria^84^, WNV^72^
*Anopheles* (*Anopheles*) *quadrimaculatus* (Say, 1824)	6,329	518	6,847	Malaria^82^, MAYV^70^, OROV^68^, WNV^85^
*Anopheles* (*Anopheles*) *walkeri* (Theobald, 1901)	27	0	27	Malaria^86^, WNV^72^
*Coquillettidia* (*Coquillettidia*) *perturbans* (Walker, 1856)	541	3	544	EEEV^68^, WNV^72^
*Culex* (*Melanoconion*) *atratus* (Theobald, 1901)	9,746	28	9,774	Unknown
*Culex* (*Culex*) *bahamensis* (Dyar & Knab, 1906)	13	0	13	WNV^72^
*Culex (Micraedes) biscaynensis* (Zavortink & O’Meara, 1999)	0	55	55	Unknown
*Culex* (*Melanoconion*) *cedecei* (Stone & Hair, 1968)	9	0	9	EVEV^68^
*Culex (Culex) coronator* (Dyar & Knab, 1906)	20,783	6,042	26,825	SLEV^68^, WNV^72^
*Culex* (*Melanoconion*) *erraticus* (Dyar & Knab, 1906)	44,601	3,122	47,723	WNV^72^
*Culex (Melanoconion) iolambdis* (Dyar, 1918)	2,263	1	2,264	Unknown
*Culex (Culex) nigripalpus* (Theobald, 1901)	1,034,119	23,366	1,057,485	EEEV^68^, EVEV^81^, KEYV^68^, ROCV^68^, SLEV^68^, WNV^72^
*Culex (Melanoconion) pilosus* (Dyar & Knab, 1906)	21	128	149	Unknown
*Culex (Culex) quinquefasciatus* (Say, 1823)	18,190	355,381	373,571	CHIKV^68^, EEEV^68^, LF, MAYV^70^, OROV^87^, ROCV^68^, SLEV^68^, WNV^72^, ZIKV^68^
*Culex* (*Culex*) *salinarius* (Coquillett, 1904)	46	7	53	EEEV^68^, WNV^72^
*Deinocerites cancer* (Theobald, 1901)	32,692	583	33,275	WNV^72^
*Mansonia (Mansonia) dyari* (Belkin, Heinemann & Page, 1970)	5,732	55	5,787	Unknown
*Mansonia (Mansonia) titillans* (Walker, 1848)	856	230	1,086	WNV^72^
*Psorophora* (*Psorophora*) *ciliata* (Fabricius, 1794)	120	2	122	WNV^72^
*Psorophora* (*Grabhamia*) *columbiae* (Dyar & Knab, 1906)	14,628	992	15,620	WNV^72^
*Psorophora* (*Janthinosoma*) *ferox* (von Humboldt, 1819)	14,198	153	14,351	MAYV^68^, OROV^68^, ROCV^79^, WNV^72^
*Psorophora* (*Psorophora*) *howardii* (Coquillett, 1901)	14	0	14	WNV^72^
*Psorophora* (*Janthinosoma*) *johnstonii* (Grabham, 1905)	19	0	19	Unknown
*Psorophora* (*Grabhamia*) *pygmaea* (Theobald, 1903)	308	35	343	Unknown
*Uranotaenia* (*Uranotaenia*) *lowii* (Theobald, 1901)	153	8	161	Unknown
*Uranotaenia* (*Uranotaenia*) *sapphirina* (Osten Sacken, 1868)	58	8	66	WNV^72^
*Wyeomyia* (*Wyeomyia*) *mitchelli* (Theobald, 1905)	4,027	6,657	10,684	WNV^83^
*Wyeomyia* (*Wyeomyia*) *vanduzeei* (Dyar & Knab, 1906)	3,245	10,273	13,518	Unknown

California Encephalitis (CALV), Chikungunya Virus (CHIKV), Dengue Virus (DENV), Eastern Equine Encephalitis Virus (EEEV), Everglades virus (EVEV), Keystone Virus (KEYV), Lymphatic Filariasis (LF), Malaria, Mayaro Virus (MAYV), Oropouche Virus (OROV), Rocio Virus (ROCV), Saint Louis Encephalitis Virus (SLEV), West Nile Virus (WNV), Yellow Fever Virus (YFV), Zika Virus (ZIKV).

### Biodiversity indices

**Figure 1:**
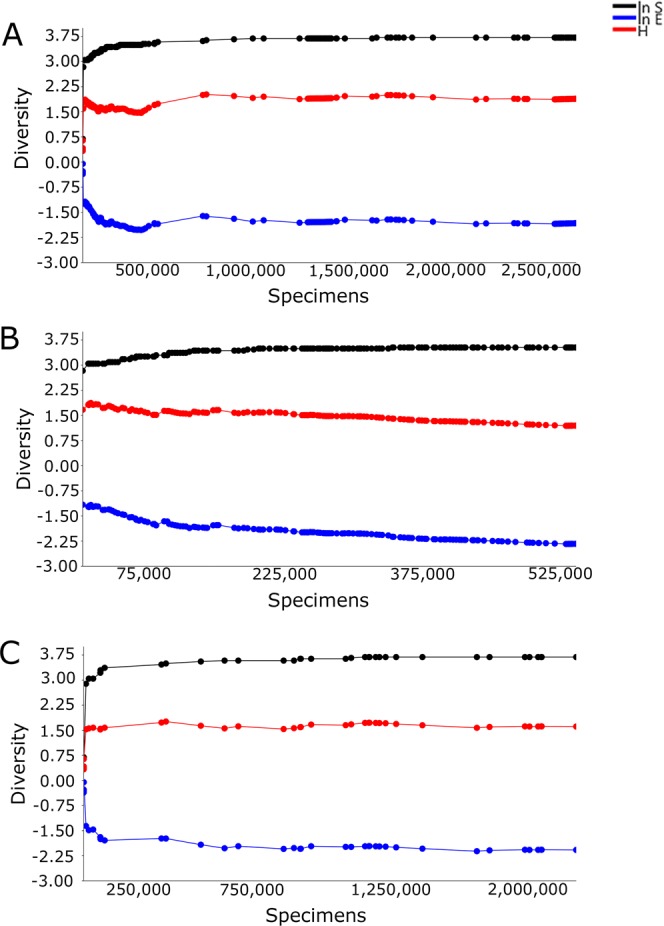
Plots of cumulative species log abundance (ln S), Shannon index (H) and log evenness (ln E) profiles (SHE) of mosquitoes collected in Miami-Dade, Florida using (**A**) BG-Sentinel and CDC traps; (**B**) BG-Sentinel traps; and (**C**) CDC traps.

Mosquito counts obtained by both BG-Sentinel and CDC traps displayed higher levels of variation for the Shannon index and log evenness. However, the log abundance remained stable (Fig. 1A). The analysis of the mosquito counts obtained by the BG-Sentinel traps pointed out many oscillations on the index values (represented by the dots in the lines), indicating higher levels of variation between samples when compared to the data obtained by the CDC traps. On the other hand, the results from the CDC traps indicated a much more heterogeneous scenario, one with considerably more variation than from the mosquitoes collected with BG-Sentinel traps (Fig. 1B).

The biodiversity indices for the CDC trap data had substantially more variation, especially with fewer samples. The log abundance yielded similar values for both BG-Sentinel and CDC traps. However, BG-Sentinel traps reached equilibrium more rapidly than CDC traps, indicating that more specimens were needed for the CDC traps to reach sampling sufficiency. Similar results were also found for the Shannon index, in which CDC traps needed more specimens to reach sampling sufficiency. However, after reaching sampling sufficiency the values remained stable, contrasting with the ones for the BG-Sentinel traps that showed a subtle but steady decline. These results may be due to the fewer number of species collected by BG-Sentinel traps. The results for the log evenness indicate that the mosquito assembly is not even for both traps, with the data from the CDC traps reaching sampling sufficiency and stabilizing, contrasting with the results from the data collected by BG-Sentinel traps, which displayed a constant decreasing trend in value (Fig. 1C).

**Figure 2:**
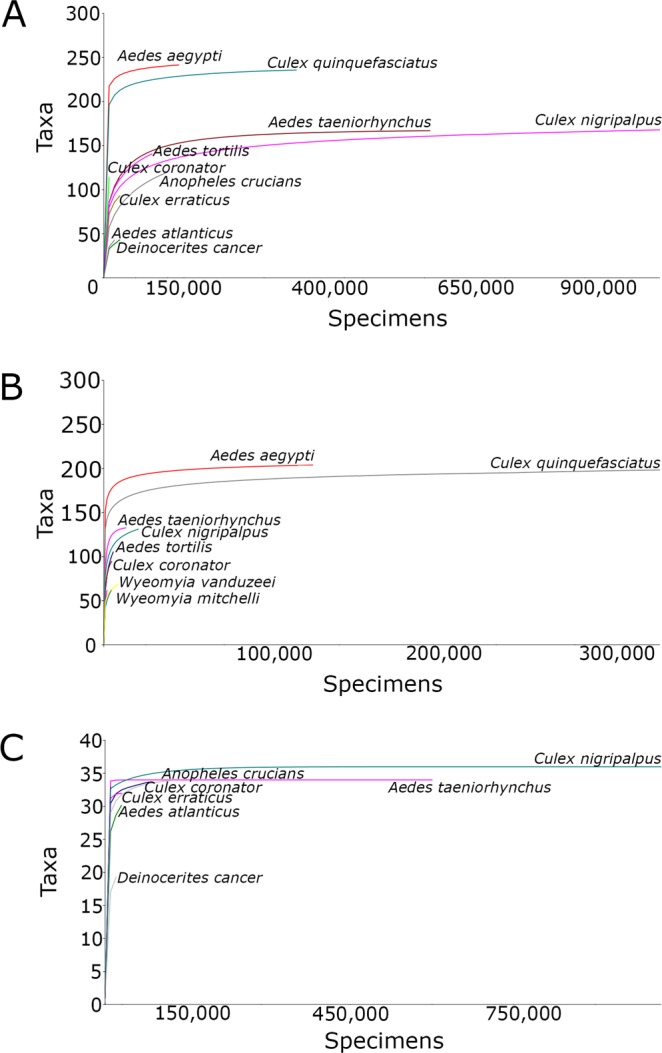
Individual rarefaction curves of mosquitoes collected in Miami-Dade, Florida. (**A**) All traps; (**B**) BG-Sentinel traps; and (**C**) CDC traps.

The individual rarefaction curves analysis considering all mosquitoes collected by both BG-Sentinel and CDC traps indicated that *Ae. aegypti*, *Ae. taeniorhynchus*, *Cx. nigripalpus* and *Cx. quinquefasciatus* yielded highly asymptotic curves (Fig. 2A). When the data from mosquitoes that were collected by BG-Sentinel traps were analyzed, only *Ae. aegypti* and *Cx. quinquefasciatus* reached the asymptote (Fig. 2B). The data obtained by CDC traps also indicated that only two species reached the asymptote, *Ae. taeniorhynchus* and *Cx. nigripalpus* (Fig. 2C). Results for the remaining species did not reach the asymptote indicating that sampling sufficiency was not achieved for these species.

### Geographical distribution and abundance of species

**Figure 3:**
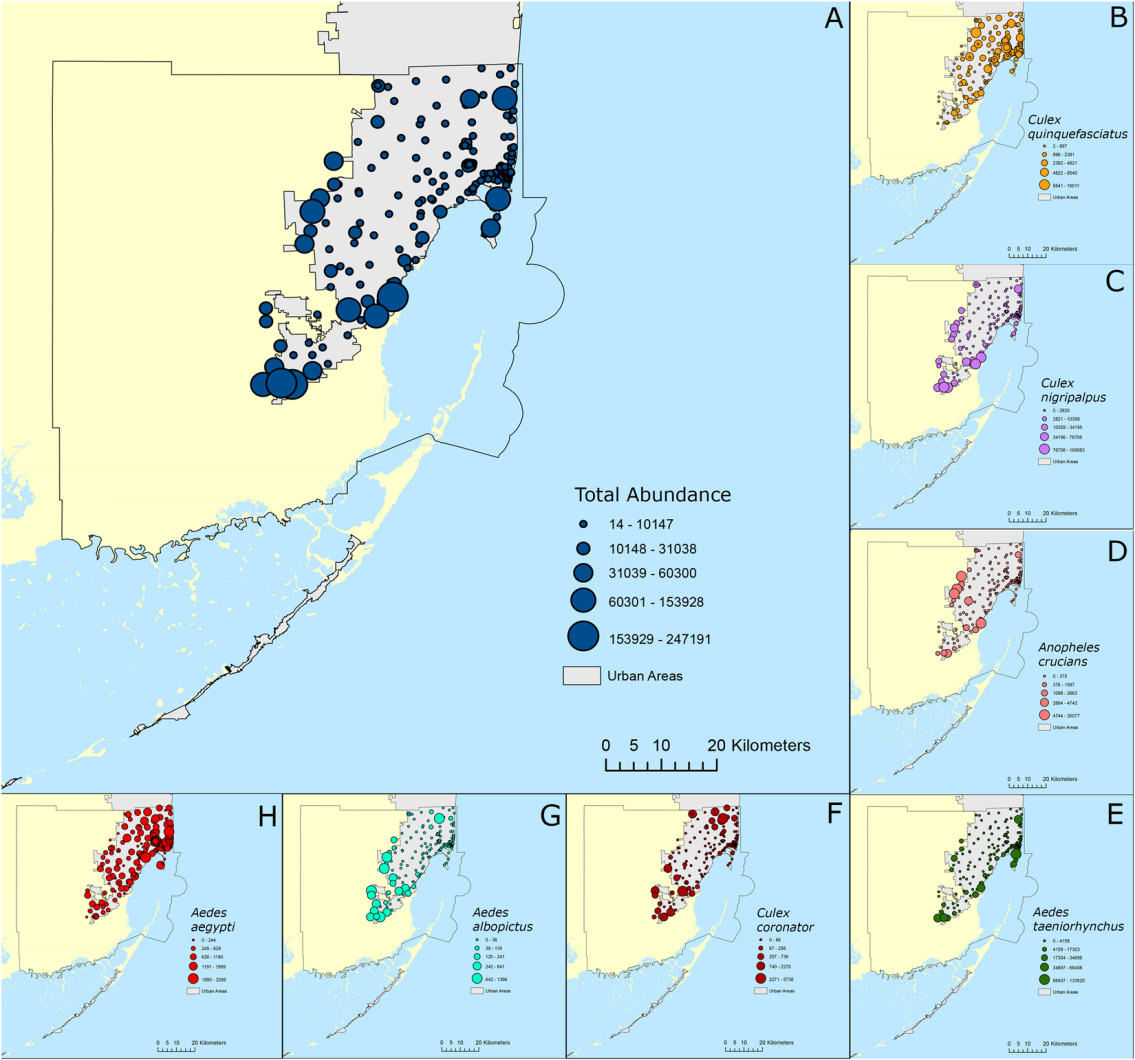
Distribution and abundance of mosquito species in Miami-Dade County, Florida. (**A**) overall abundance; (**B**) *Culex quinquefasciatus*; (**C**) *Culex nigripalpus*; (**D**) *Anopheles crucians*; (**E**) *Aedes taeniorhynchus*; (**F**) *Culex coronator*; (**G**) *Aedes albopictus*; (**H**) *Aedes aegypti*.

The overall presence and abundance of mosquitoes were found to be higher near the Everglades and the coast than in the core of the city where urbanization is more intense (Fig. 3A). However, when species were analyzed individually a broader spectrum of variation was found. *Culex quinquefasciatus* was distributed in high numbers throughout the urbanized areas regardless of their location indicating that this species is well adapted to the urban environment of Miami-Dade County (Fig. 3B). *Culex nigripalpus*, on the other hand, was shown to be more limited to the border of the urban areas, not commonly found in urbanized areas (Fig. 3C). A similar result was found for *An. crucians*, in which this species was more abundant in areas bordering the limits of the urbanized areas of the Miami-Dade County (Fig. 3D). *Aedes taeniorhynchus* was collected in high numbers in areas near its natural habitats, including mangrove remnants in urbanized areas such as in the Virginia Keys and Miami Beach (Fig. 3E). *Culex coronator* was collected in relatively high numbers throughout the whole Miami-Dade County, including urbanized areas (Fig. 3F). As expected, *Ae. albopictus* was more abundantly found in areas at the border of the urban areas, not being commonly found in highly urbanized areas (Fig. 3G). *Aedes aegypti*, on the other hand, was the most prevalent species collected in Miami-Dade County, being abundantly collected throughout the urban areas of Miami-Dade County (Fig. 3H).

### Seasonal variation of species

**Figure 4:**
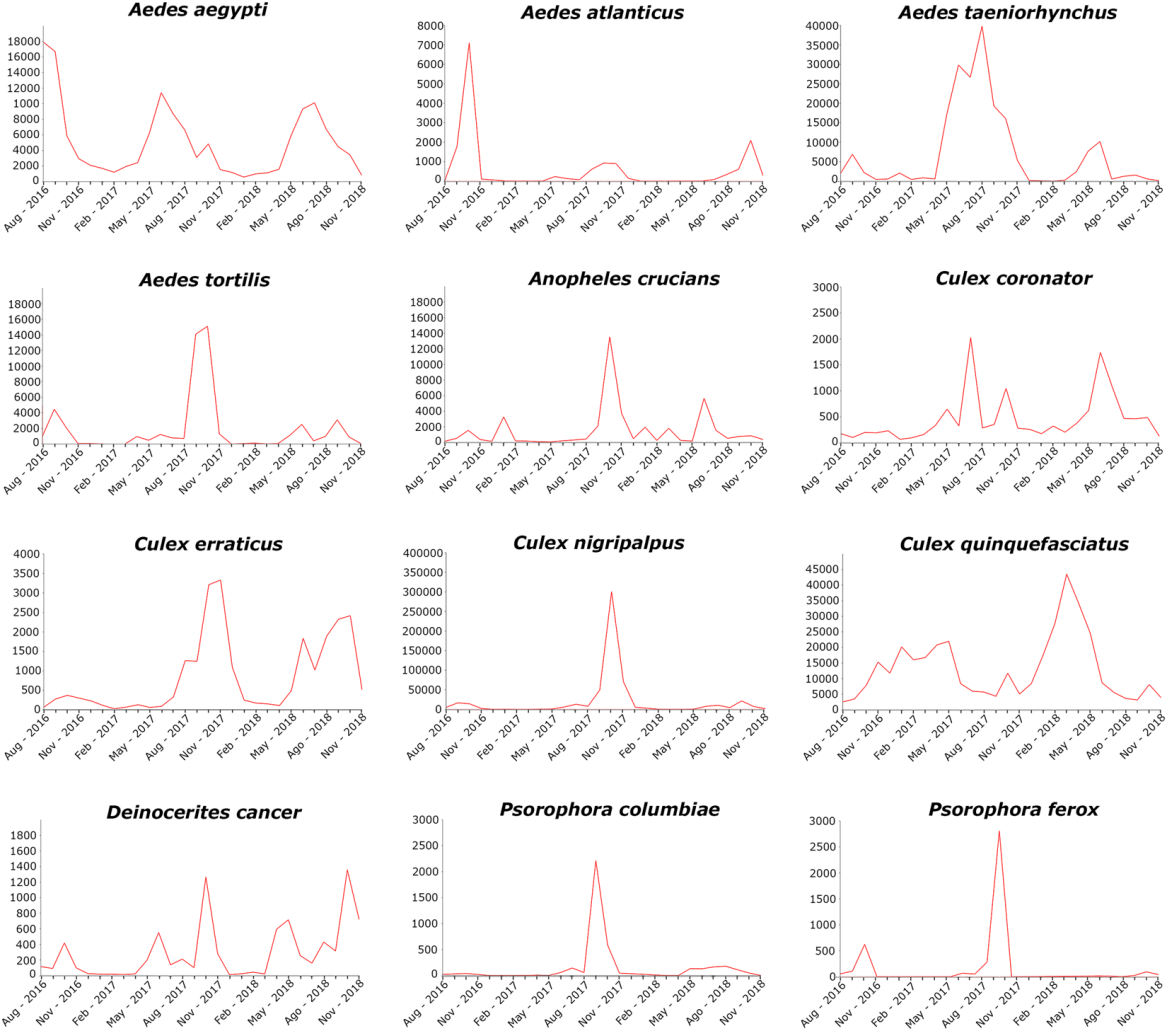
Seasonal variation of mosquito species in Miami-Dade County, Florida.

The seasonal variation varied greatly accordingly to species. However, from a broader perspective, there were essentially two main patterns, species that peaked in abundance during the warmer months of the year and others during the colder. Unexpectedly, most species had their peak abundance in November when temperatures are considerably cooler than during the peak of the summer in August. On the other hand, *Ae. aegypti*, *Ae. taeniorhynchus* and *Cx. coronator* had their peak abundance during the summer. *Culex quinquefasciatus* showed a unique pattern, being more abundant during the winter and decreasing in numbers during the summer. *Aedes aegypti* and *Cx. quinquefasciatus* were collected in high numbers in all months of the year, with the lowest number of specimens collected being 585 *Ae. aegypti* in January 2017, and 2,515 *Cx. quinquefasciatus* in August 2016 (Fig. 4).

## Discussion

Urbanization processes are often responsible for decreasing the richness of species, followed by the increase in abundance of the few species that are able to endure and thrive in urban environments^34–36^. This process may lead not only to local adaptation of vector mosquitoes^26,37–41^, but also can profoundly affect the ecology and behavior of species that are not fully adapted to urban environments^42^.

Our results revealed that the mosquito community in Miami-Dade County, Florida is comprised of five highly dominant species. Of the five most abundant species, *Cx. nigripalpus, Cx. quinquefasciatus, Ae. aegypti* and *An. crucians* are primary vectors of arboviruses. *Aedes aegypti* and *Cx. quinquefasciatus* were abundantly found throughout Miami-Dade County regardless of urbanization level, vegetation or socioeconomic variations. To a lesser extent. *An. crucians*, *Cx. coronator* and *Cx. nigripalpus* were also widely distributed,

Furthermore, *Ae. aegypti* and *Cx. quinquefasciatus* were abundant year-round, indicating that these species are well adapted to thrive in the urban environments of Miami-Dade County. *Culex coronator* also had a relatively high abundance and is increasingly becoming a public health concern. Growing evidence shows that *Cx. coronator* is becoming more adapted to thrive in urban environments and, as a consequence, it is increasing in presence and abundance in urban areas^43^. *Culex coronator* was reported in the U.S. in 2004 in Louisiana^44^. Since then, it has spread and has been commonly found in most of the Southeastern states including Mississippi, Alabama, Florida and Georgia. In 2017, *Cx. coronator* was detected in the state of Tennessee, where it is currently considered an established species^45,46^.

SHE profiles reached equilibrium after a rapid initial variation. This indicates that the cumulative species log abundance, Shannon index and log evenness are representative of the community composition and abundance of mosquitoes, and moreover that the sampling effort was adequate. On the other hand, despite the high number of traps and more than two years of weekly collections, the individual rarefaction curves analysis indicated that only *Ae. aegypti*, *Ae. taeniorhynchus*, *Cx. nigripalpus* and *Cx. quinquefasciatus* reached sampling sufficiency with substantial degrees of confidence for predicting the expected presence of those species for smaller sample sizes. The remaining species did not reach the asymptote and therefore were not a representative measure of the completeness of sampling indicating that their presence and abundance may have been underestimated. These species were found in relatively low densities and were locally concentrated in specific habitats, leading to an increased variance in their sampling. The underestimation of vector species should be of concern since many of the species found in Miami-Dade County were previously implicated in the transmission of arboviruses in the state of Florida^28–30,43,47^.

Our results imply that an increase in the surveillance efforts, such as the inclusion of immature surveys should be considered in addition to the current surveillance system. The combined immature and adult mosquito surveillance methods are complementary and would help detect mosquito species that are not always successfully detected solely by the collection of adult mosquitoes.

Overall, our results suggest that the most abundant mosquito species present in Miami-Dade County are well adapted to thrive in urban environments, or are becoming more adapted to it. Furthermore, the intensification in urbanization processes have resulted in the increased proximity of residential areas to natural areas, therefore, increasing the contact between humans and naturally occurring native sylvatic mosquitoes, such as the aggressive *Ae. taeniorhynchus*, as well as creating more suitable habitats for mosquito species adapted to thrive in urban environments.

Controlling mosquito populations is becoming more problematic due to, among other things, the increase in the levels of insecticide resistance and lack of effective new control tools^22,48^. In this context, Miami-Dade County is exceptionally vulnerable to the introduction of arboviruses and subsequent outbreaks, serving as a gateway for the introduction and spread of arboviruses to the U.S.

The introduction of arboviruses inadvertently carried by human movements into and from endemic areas is unavoidable^49–53^. For example, in 2017, around 4 billion people were transported globally by airlines, with the U.S. alone being responsible for transporting 719 million people in domestic flights and 104 million in international flights^54,55^. Brazil receives around 2 million tourists from Europe every year, with many of them visiting areas with ongoing active VBD transmission. In 2018 only, several cases of YFV were reported among European travelers returning from Brazil to Europe, including two deaths^56,57^.

*Aedes aegypti* has been proven an excellent vector of YFV^58^, and there is growing evidence that *Ae. albopictus* is a competent vector, thus important for the maintenance of the YFV cycle in suburban and urban areas^59^. Furthermore, specimens of *Ae. albopictus* were found naturally infected with YFV in a transmission hotspot in Brazil corroborating its importance in the patterns of YFV transmission^60^. Evidence points to a single introduction of YFV in the Southeast region of Brazil to be responsible for the current outbreak^61^. Furthermore, *Ae. aegypti* and *Ae. albopictus* from Brazil and Florida are similarly competent to transmit arboviruses^62^, exposing the high-risk scenario for Florida residents and tourists.

Continued surveillance, public education, environmental ordinance, and active control of mosquito populations are critical for the prevention of VBD outbreaks. The current scientific consensus is pointing to the increased risk of VBD outbreaks due to many factors, including human behavior and global warming, highlighting even more the need for better mosquito control strategies and an increase in awareness of the general public to the needs for well-established mosquito control operations.

This study serves as a stepping stone for future studies that are needed to uncover the population dynamics patterns of the mosquito species present in Miami-Dade and to assess the risk they pose to both residents and tourists. In our opinion, future studies should focus three main foci: (i) determine how populations of mosquito vector species are largely regulated by the availability of essential resources for their survival in habitats at the local neighborhood level; (ii) Investigate why several recently introduced invasive vector mosquito species such as *Culex coronator* and *Culex panocossa*, and historically less common vector species are now locally abundant in habitats in the urban built environment of Miami-Dade County; and (iii) determine how the effectiveness of different vector control methods, considering the development of resistance to insecticides by vector mosquitoes, varies in urban habitats, and how the proliferation of vector mosquitoes can be locally reduced by targeting or modifying specific features of the urban environment.

Public health attention should not be diverted from finding, monitoring and controlling the major known mosquito vectors, but extended towards the neglected and invasive species as well. Furthermore, continuous monitoring is mandatory for effectively guiding mosquito control operations.

## Methods

Miami-Dade is the most populous and third largest County in Florida. It has almost 3 million people and more than 6 million km^2^, spreading from the Everglades on the west to the Biscayne Bay on the east^63^. The surveillance grid was designed to have at least one trap per 1.6 km^2^ in the urbanized areas of Miami, as well as traps at the city limits bordering the Everglades. Additional traps were also deployed in points of interest such as touristic areas, shopping malls and areas with outdoor activities, where residents and tourist would be more exposed to mosquitoes.

**Figure 5:**
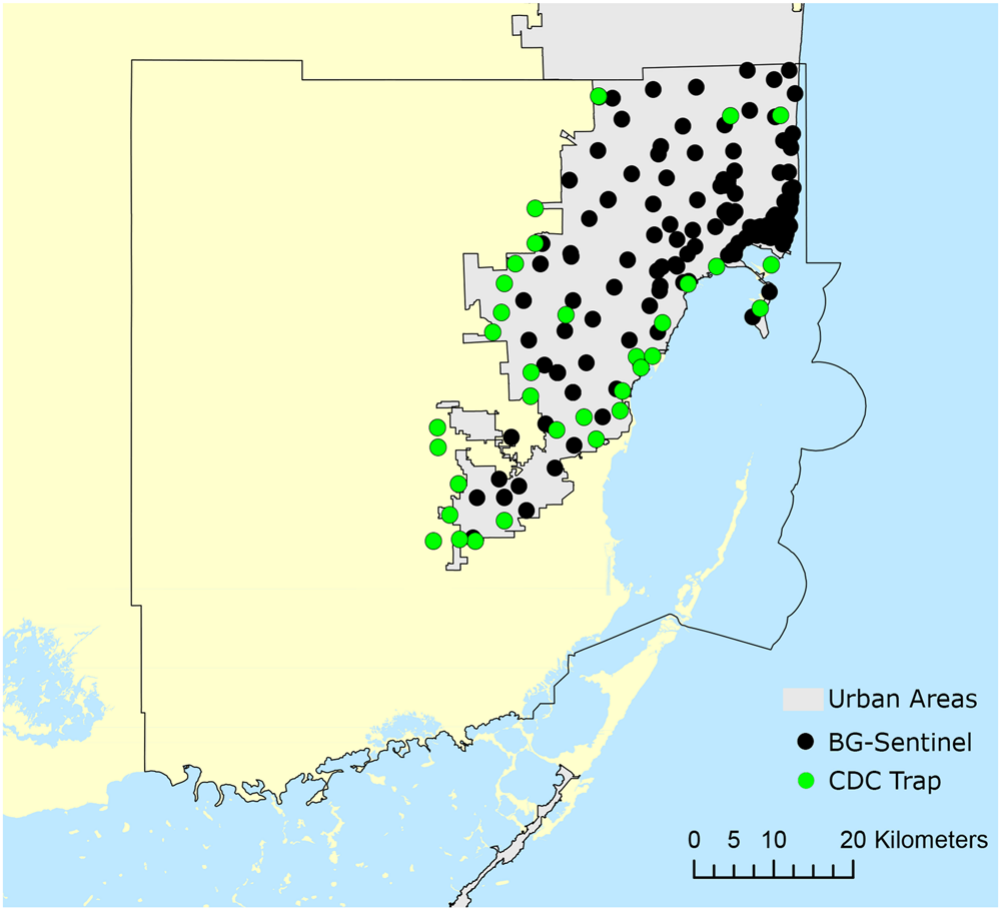
Map of Miami-Dade County, Florida. Urban areas are displayed in gray, CDC traps in green and BG-Sentinel traps in black.

The Miami-Dade County Mosquito Control surveillance grid is comprised of 191 traps, being 157 BG-Sentinel (Biogents AG, Regensburg, Germany) and 34 CDC traps (Fig. 5). Each trap was deployed weekly for 24 hours from August 2016 to November 2018. All traps were baited with CO_2_ using a container filled with 1 Kg of dry ice pellets. All collected mosquitoes were transported to the Miami-Dade County Mosquito Control Laboratory and subsequently morphologically identified using taxonomic keys^64^. The surveillance database does not include data of male mosquitoes since both BG-Sentinel and CDC traps mainly attract females seeking for blood feeding and, therefore, male mosquitoes collected were considered accidental catches and were not considered informative for surveillance purposes.

Since this study posed less than minimal risk to participants and did not involve endangered or protected species the Institutional Review Board at the University of Miami determined that the study be exempt from institutional review board assessment (IRB Protocol Number: 20161212).

Analyzes were carried out for all collected mosquitoes, and subsequently sub-setted for mosquitoes collected using BG-Sentinel and CDC traps. To compare mosquito diversity in samples with different sizes, to provide an estimation of the number of species in samples with fewer specimens and to estimate sampling sufficiency, analyzes of individual rarefaction curves were carried out. Subsequently, plots of cumulative profiles of species log abundance (ln S), Shannon index (H) and log evenness (ln E) (SHE) were calculated for all samples. This model calculates the ln S, H and ln E values individually for each sample, repeating the process for the next sample and so on consecutively until the last one. The results can be interpreted based on deviations from the straight line and are useful to help to grasp subtle trends in species composition and variations in the mosquito assembly^65^.

Analyses were carried out with 10,000 randomizations without replacement and a 95% confidence interval using Past software (v.3.16)^66,67^. Figures 1 and 4 were produced using ArcGIS (v.10.2) using maps freely available at www.census.gov.
